# Animals, *Quo Vadis*? Welcome to a New, Multidisciplinary, Integrated, Open Access Journal: *Animals*

**DOI:** 10.3390/ani1010001

**Published:** 2010-09-24

**Authors:** Clive Phillips

**Affiliations:** *Founding Editor-in-Chief of Animals*, Center for Animal Welfare and Ethics, School of Veterinary Science, University of Queensland, Gatton Campus, Queensland 4343, Australia; E-Mail: c.phillips@uq.edu.au; Tel.: +61 7 5460 1158

Without animals this planet would be a very different place, indeed many of the remaining life forms could not exist. As animals ourselves we are linked to a vast network of moving, living, reproducing organisms that form an essential part of the various ecosystems that are themselves competing for survival. By virtue of our large cognitive capacity and complex societal living structures, we manage and influence many of these ecosystems, in the most extreme way by keeping animals captive for our own benefit. For these captive animals we manage their health, nutrition, reproduction and even their genetic composition; for wild animals we often have major influences on the habitat, and even the animals themselves, that affect their viability. This intricate interdependence of humans on other animals is increasingly difficult to sustain in the face of a growing human population, diminishing natural resources, accelerating changes in climate and rapidly evolving societal standards. The continued existence of many animal species cannot be guaranteed, some systems of keeping captive animals are judged to be immoral, and even within widely accepted systems individual animals may be subjected to suffering. Captive animals are suspected of widespread transmission of diseases, contributing to climate change and degradation of the landscape. Changing dietary habits to include more animal products are placing livestock production systems under strain worldwide. Conversely, animals feed us, clothe us, protect us, amuse us, some probably even reciprocate the love that we give to them, and many people in developing countries are reliant on their livestock. Evaluating animals' place in the world today is even more essential than ever before.

Resolving animal issues cannot just be by scientific endeavor, but requires strategic decisions to be made by the whole community. Providing information rapidly and to everyone is an essential part of empowering the community and its leaders to take the necessary decisions in an informed manner. This new journal, *Animals*, will provide a forum for all to become rapidly familiar with the latest scientific research on animals and their place in different ecosystems. The research may be fundamental or applied, and could use a range of techniques from molecular to the study of animal systems. Rapid review and publication will enable scientists to act much more quickly to address the concerns outlined above. Long publishing delays of more than one year that hinder your grant and job applications can be avoided by using this journal, which will publish papers as soon as the peer review process has been completed and editorial requirements met. Due to their widespread availability, open access journal articles are increasingly likely to be cited, and several of the established MDPI journals have already been awarded Impact Factors that are as high as many of the leading animal science journals. The page charges for *Animals* will be modest, in comparison with both other open access journals and the full cost of the research, and are waived for well-prepared contributions received in 2010 and 2011. For most publicly-funded research, open access is a *right* for the community members because the work has been funded, indirectly, from the taxes that they pay to the government. For the researcher, open access gives the opportunity to expose their work to a broader community than just their scientific peers, allowing them to gain from the vastly increased contact with those who will ultimately benefit from the research. The publisher MDPI has 14 years of open access publishing experience and, with its base in Switzerland, is able to offer a reliable and reputable publishing forum. This journal has an august body of Editors, with a wide range of experience that will allow them to accurately appraise publications in a wide range of animal topics.

In recent years, the range of topics covered in animal research has expanded considerably, from animal genetics, with its rapidly developing methodology and potential for change, to animal emotions, whose very existence used to be denied. In *Animals* it will be possible, and often desirable, to include video footage—for example, see the footage from a study of feeding behavior in the endangered mahogany glider as a supplementary file for this editorial—to support the arguments made in the scholarly, rigorous articles that will describe animals' place in the world today. Color figures can also be included at no extra cost, for example, Figure 1 below, which illustrates intensities of noxious gases that colleagues and I recently measured in sheep accommodation.

Another advantage of electronic publishing is that articles are not limited by length, and authors are encouraged to provide adequate detail to enable the study to be fully comprehended, and extended if necessary, and the results to be utilized in further studies, for example in meta-analyses. Notwithstanding this, authors are also encouraged to write succinctly and to provide a summary of their article in a form that is suitable for an open access forum.

The journal will publish regular research papers, reviews of books and topical issues, communications, and short notes. Ideas for special issues are welcomed, for example from conferences addressing a topical animal theme. Researchers are invited to submit high quality, original articles and erudite reviews that will help us to improve our understanding of the place of animals on our planet, and ultimately to benefit the lives of animals, and ourselves.

## Figures and Tables

**Figure 1:**
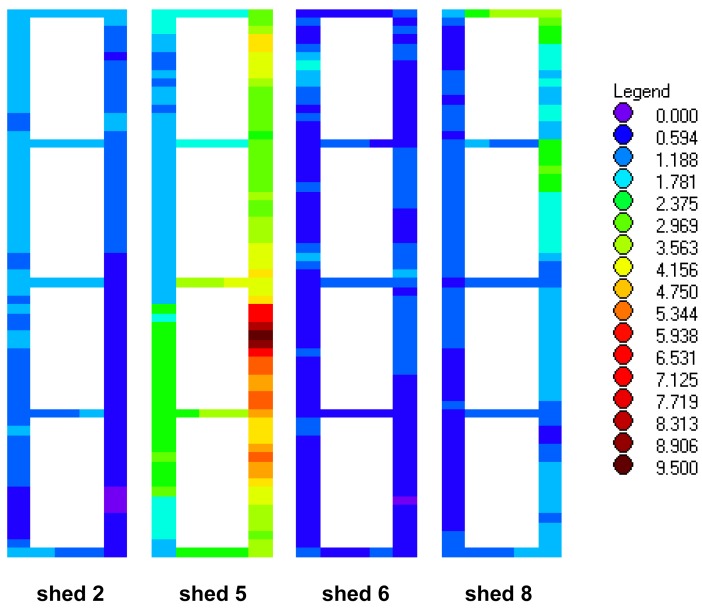
Ammonia concentrations in four sheep buildings. Mean ammonia concentrations mapped at 2 m intervals (n = 48) [1].

## Supplementary Files

Supplementary File 1
